# Lumbar Pneumorrhachis Associated With Basilar Skull Fractures

**DOI:** 10.7759/cureus.19703

**Published:** 2021-11-18

**Authors:** Miner Ross, Maryam N Shahin, Brannan E O'Neill, Jesse J Liu

**Affiliations:** 1 Neurological Surgery, Oregon Health & Science University, Portland, USA

**Keywords:** traumatic csf leak, neurotrauma, pneumorrhachis, skull fracture

## Abstract

Lumbar pneumorrhachis following head injury is rare and commonly asymptomatic but can be indicative of skull fracture and cerebrospinal fluid (CSF) leak, which may warrant intervention. A PubMed review of the literature was performed using a keyword search to identify cases examining lumbar pneumorrhachis following head injury. Our case series included two patients who had lumbar pneumorrhachis between September 2019 and May 2020 at our center. The literature review summarizes 16 patients from 14 prior reports of pneumorrhachis. In our two-patient case series, neither patient required direct intervention for either pneumorrhachis or CSF leak. Pneumorrhachis is rare following an isolated head injury and is associated with basilar skull fractures and CSF leak. Pneumorrhachis should alert clinicians to the possibility of a CSF leak, which may require intervention.

## Introduction

Background

Pneumorrhachis is the presence of air within the spinal canal, and can be either epidural or intradural [1-8]. Pneumorrhachis is most commonly caused by the direct introduction of air into the spinal canal, typically due to penetrating trauma or iatrogenically via surgery [2, 3, 5-9]. Traumatic pneumorrhachis secondary to isolated head injury is rare, with only limited case reports in the literature. Of these reports, the majority report pneumorrhachis within the cervical spine only; pneumorrhachis is seldom found within the thoracolumbar spine. Here we provide a comprehensive systematic review of pneumorrhachis due to isolated head injury and two case reports of pneumorrhachis extending to the lumbar spine in this setting.

Methods

A search of the PubMed database maintained by the US National Library of Medicine was undertaken for all articles published through 2019 related to pneumorrhachis. This included, among other search terms; “spinal pneumatosis”, “spinal pneumocele”, “spinal emphysema”, “intraspinal air”, “air myelogram”, “pneumomyelogram”, and “pneumosaccus”. All articles were retrieved in their full-text format and analyzed for their appropriateness for inclusion. References within these articles were also reviewed to identify other case reports. Cases of direct spinal trauma, iatrogenic cases, and cases with other causes of pneumorrhachis were excluded.

## Case presentation

Case 1

**Figure 1:**
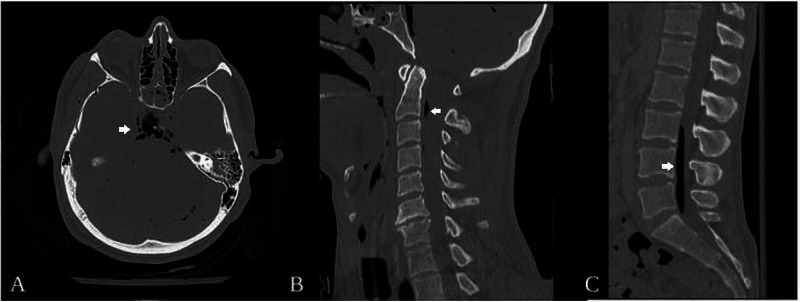
Case 1: Representative Computed Tomography (A) non-contrast head CT axial image demonstrating diffuse pneumocephalus, (B) non-contrast cervical spine CT sagittal reconstruction showing air at the level of C2, and (C) non-contrast lumbar spine CT sagittal reconstruction showing air extending from L3-L5. (CT: computed tomography.)

This is an otherwise healthy 35-year-old male who was brought to our emergency department by ambulance after being crushed underneath a car; while performing some repairs on the undercarriage the jacks failed and the vehicle fell directly onto his head. Emergency personnel at the scene reported he was alert and coherent, but had obviously sustained significant facial trauma and he was intubated for airway protection. On arrival to our institution, head, spine, chest, abdomen, and pelvis computed tomography (CT) scans were obtained in accordance with standard trauma protocols. Multiple, comminuted facial and basilar skull fractures, with a large volume of pneumocephalus, though without any evidence of intracranial hemorrhage, were found (Figure 1A). Spinal column imaging revealed intradural air spanning C1-C4 and L3-S1 levels (Figure 1B and 1C). There was no imaging evidence of direct spinal trauma, nor any evidence of pneumothorax or pneumoperitoneum.

Neurologic examination revealed an intubated young male with a Glasgow Coma Scale (GCS) of 8T, given that he opened his eyes briefly when stimulated, and displayed purposeful and localizing movements in all extremities but did not follow commands. He had no apparent focal neurologic deficits. He had multiple facial ecchymoses and lacerations as well as dried blood at the nares and in both ears, though without obvious rhinorrhea or otorrhea. In accordance with institutional policy, head-injured patients with depressed GCS are admitted to the trauma intensive care unit (ICU) and head imaging is repeated after 6 hours. In this case, a second head CT was unchanged from the first. His GCS improved rapidly and he was extubated 9 hours after admission. Repeat neurologic examination revealed delayed development of a right facial nerve paresis for which he was prescribed a two-week course of dexamethasone without significant improvement noted by the time of discharge. He otherwise remained without strength or sensation deficits in his extremities. He was discharged home in good condition on post-injury day 3.

Case 2

**Figure 2:**
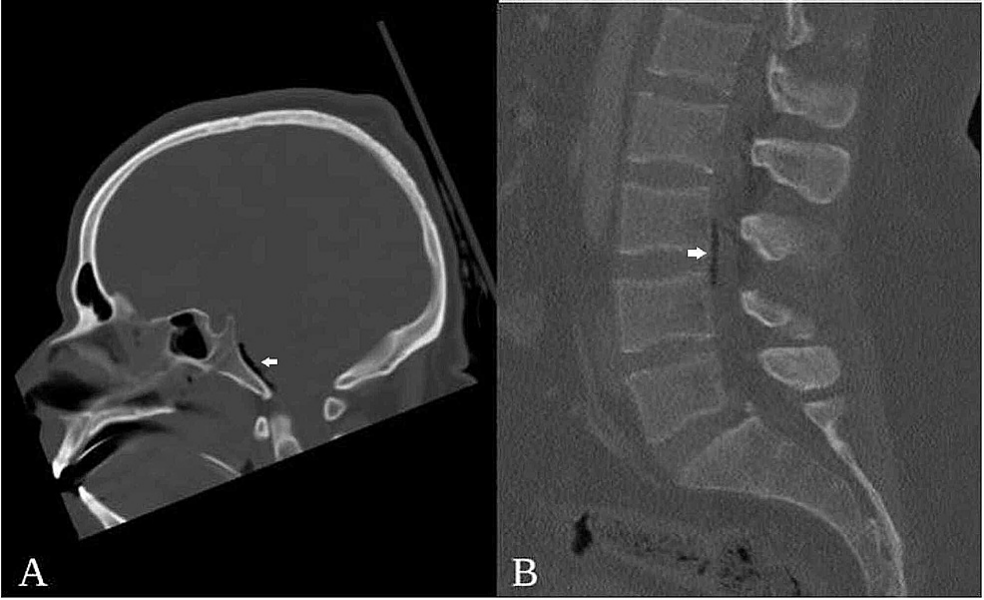
Case 2 Representative Computed Tomography (A) non-contrast head CT sagittal reconstruction demonstrating pneumocephalus at the level of the clivus, and (B) non-contrast lumbar spine CT sagittal reconstruction showing air at the L3-L4 levels. (CT: computed tomography)

This is an otherwise healthy 25-year-old male who was brought to our emergency department by ambulance after a fall of 8-10 meters; while leaning against the railing of a third-story balcony he lost balance and fell backward over the rail to the ground below. Emergency personnel at the scene found him comatose and pulseless. After several rounds of cardiopulmonary resuscitation (CPR) and rapid sequence intubation, he was stabilized for transport to our hospital. On arrival to the emergency department, he had developed refractory hypotension and lost a carotid pulse for which he again required a round of CPR before return of spontaneous circulation. Once resuscitated and stabilized, head, spine, chest, abdomen, and pelvis CT scans were obtained. He was found to have bilateral temporal bone fractures as well as a transverse clival fracture, as well as diffuse but predominantly right-sided traumatic subarachnoid hemorrhages with scattered locules of pneumocephalus (Figure 2A). Spinal imaging revealed intradural air at the L3-L4 levels (Figure 2B). There was no imaging evidence of direct spinal trauma, nor any evidence of pneumothorax or pneumoperitoneum.

Neurological examination on arrival revealed an intubated young male with a GCS of 6T: he did not open his eyes nor display any localizing movements but was able to withdraw all of his extremities from painful stimuli. He had dried blood in both ears but was otherwise without otorrhea or rhinorrhea.

He was admitted to our trauma ICU and an intracranial pressure monitor was placed and monitoring was noted as normal. A follow-up head CT was unchanged. His GCS improved rapidly and he was extubated 11 hours after admission, and his intracranial monitor was removed. Repeat neurological examination noted that he was coherent and oriented, but amnestic to his injury. He had developed a left facial nerve paresis and was prescribed a two-week course of prednisone without significant improvement noted by the time of discharge. He remained without strength or sensation deficits of his extremities. Pelvic injuries necessitated operative fixation on hospital day 2. He was discharged to rehabilitation in good condition on post-injury day 8.

## Discussion

Following a detailed PubMed search we reviewed 14 published case reports comprising 16 total cases of pneumorrhachis associated with head injury without direct spinal injury. These 16 cases, in addition to our two cases, are summarized in Table 1 [1-8, 10-15].

**Table 1:** Summary of Reported Cases of Pneumorrhachis After Isolated Head Injury GCS: Glasgow coma score; CSF: cerebrospinal fluid

Author (Year)	Age (years) / Sex	Mechanism	GCS on Admission	Pneumorrhacchis Location	Other Findings	CSF leak	Interventions	Outcome
Gordon & Hardman (1977) [11]	20 / M	Motor vehicle crash	4	Cervical	Frontal and parietal skull fractures, pneumocephalus, blunt cerebrovascular injuries	No; however, bloody otorrhea / rhinorrhea noted	None	Death 14 days post-injury
Newbold et al. (1987) [5]	24 / M	Motor vehicle crash	Awake	Cervical	Depressed calvarial fractures, sphenoid sinus fracture, pneumocephalus	CSF rhinorrhea	Skull fracture elevation, transsphenoidal CSF leak repair	Alive but still hospitalized at 2 months
Mangiardi et al. (1987) [4]	63 / M	Assault with blunt trauma	13	Cervical	Depressed occipital and temporal skull fractures, pneumocephalus	CSF otorrhea	Lumbar subarachnoid drainage, operative CSF leak repair	CSF leak resolved, left facial nerve paralysis
Prins & Vencken (1989) [13]	40 / M	Fall down stairs	Comatose	Lumbar	Skull base fractures, cerebral contusions, pneumocephalus	Not described	Not described	Not reported
Yip et al. (1990) [14]	40 / M	Assault with blunt trauma	15	Cervical	Frontal sinus fracture	None	Antibiotics	Discharged well after uneventful observation
Day et al. (1994) [10]	70 / M	Fall down stairs	7	Cervical	Temporal bone fracture subdural hematoma, cerebral contusions, pneumocephalus	No; however, bloody otorrhea noted	None	Death 2 days post-injury
Sinha & Mantle (2000) [7]	21 / M	Motor vehicle crash	5	Cervical	Frontal and basilar skull fractures, cerebral contusions, pneumocephalus	CSF rhinorrhea	None	Brain death 40 hours post-injury
Inamasu et al. (2002) [12]	16 yo M	Bicycle crash	Conscious	Cervical	Temporal bone fracture, pneumocephalus	CSF otorrhea	Lumbar subarachnoid drainage	Discharged well 30 days post-injury
Çayli et al. (2003) [1]	17 / M	Fall from height	8	Cervical, lumbar	Pneumocephalus	CSF otorrhea	Antibiotics	Discharged well 10 days post-injury
	38 / F	Fall from height	10	Cervical	Pneumocephalus	CSF otorrhea	Antibiotics, lumbar subarachnoid drainage	Discharged well 15 days post-injury
Yousaf et al. (2003) [8]	44 / M	Fall from standing	13	Cervical	Temporal bone fracture, pneumocephalus, C4 and C8 radiculopathies	CSF otorrhea	None	Neurologically improved at 48 hours, CSF leak resolved within 5 days
Oertel et al. (2006) [6]	51 / F	Motor vehicle crash	4	Cervical	Displaced skull fracture, temporal bone fracture, sphenoid sinus fracture, subdural hematoma, subarachnoid hemorrhage, pneumocephalus	Not described	None	Brain death
Coskun et al. (2009) [2]	49 / M	Motor vehicle crash	14	Cervical, thoracic, lumbar	Multiple calvarial and sinus fractures, epidural hematoma, subdural hematoma, subarachnoid hemorrhage, pneumocephalus	Not described	None	Fully recovered at 4 months
Hadjigeorgiou et al. (2016) [3]	26 / M	Motor vehicle crash	3	Cervical	Basilar skull fracture, subarachnoid hemorrhage, cerebral edema, pneumocephalus	Not described	None	Death 12 days post-injury
	39 / M	Motor vehicle crash	7	Cervical	Basilar skull fracture, subdural hematoma, cerebral contusions, subarachnoid hemorrhage, cerebral edema, pneumocephalus	Not described	Intracranial pressure monitoring, secondary decompressive hemicraniectomy	Death 8 days after craniectomy (11 days post-injury)
Yuce et al. (2016) [15]	25 / M	Motor vehicle collision	Confused	Lumbar	Pneumocephalus	Not described	Not described	Not reported
Present Study	35 / M	Crush injury	8	Cervical , lumbar	Basilar skull fractures, pneumocephalus, facial nerve palsy	None	None	Discharged stable 3 days post-injury
	25 / M	Fall from height	6	Lumbar	Basilar skull fractures, subarachnoid hemorrhage, pneumocephalus, facial nerve palsy	None	Intracranial pressure monitoring	Discharged stable 8 days post-injury

In 1977, Gordon and Hardman first reported pneumorrhachis in a trauma patient in which multiple skull fractures were presumed to have led to the presence of air in the cervical spine [11]. They hypothesized that head-down positioning used by emergency medical personnel in resuscitation allowed air to migrate caudally to the cervical subarachnoid space. The first known traumatic lumbar pneumorrhachis was reported in 1989 by Prins and Vencken [13]. They described in a comatose 40-year old male who presented post-trauma and was found to have basilar skull fractures with an air-fluid level in the lumbar thecal sac; they similarly hypothesized that head-down positioning following development of traumatic pneumocephalus allowed air to migrate caudally. A report by Day et al. in 1994 [10] of a patient who fell down stairs and was found to have cervical pneumorrhachis describes the same mechanism, as does a report by Sinha and Mantle in 2000 [7] of a patient involved in a severe motor vehicle crash. Sinha and Mantle additionally cautioned against the use of nitrous oxide to avoid expansion of air-filled pockets and thus development of tension pneumocephalus, as well as describing that the findings of pneumorrhachis and/or pneumocephalus should be viewed as a contraindication to positive-pressure ventilation [7]. Oertel et al. specifically make reference to their case report of the patient being intubated after a prolonged interval of mask ventilation [6].

In both cases in our case series, patients were intubated at the scene of injuries, likely after some period of positive-pressure mask ventilation. We hypothesize that this entrained sufficient intradural air to account for imaging findings.

In a number of reports, cervical pneumorrhachis visualized on plain films was described as a secondary sign of basilar skull fractures and proposed as a cue to obtain cross-sectional cranial imaging [1, 5, 7, 10, 12, 14]. Today, CT imaging is widely available and is the first diagnostic modality employed in the workup of the vast majority of trauma patients. This likely explains why the phenomenon was not reported until 1977, and why the frequency and number of case reports has increased over time. Skull fractures are reported in 15 of the 18 known cases, with the other three reports not noting cranial imaging findings. In the absence of penetrating spinal trauma or other injuries (e.g. pneumothorax, pneumoperitoneum), which could entrain air into the spinal canal, the presumed mechanism of all subarachnoid pneumorrhachis is secondary to skull fracture.

Eroglu et al. reported a non-traumatic case of spontaneous lumbar pneumorrhachis which was symptomatic via back and leg pain [9]. Multiple cases of symptomatic pneumorrhachis secondary to either direct spinal trauma or iatrogenic air have been reported, with the majority being secondary to insertion of epidural anesthetic catheters [16-20]. In the literature review we have compiled, only Yousaf et al. [8] report a case in which the air locule was known to be symptomatic per se; the patient had apparent C4 and C8 radiculopathies in the setting of long-segment cervical pneumorrhachis. Many of the other reported cases, however, presented with depressed GCS and may have been unable to report such symptoms. Both patients in our series suffered facial nerve injuries secondary to fractures of the petrous temporal bone, but no neurologic sequela attributable to intracranial or intraspinal air. In the case reported by Mangiardi et al., a facial nerve paralysis was also noted [7].

In seven previously reported cases, the finding of pneumorrhachis was associated with a clinically apparent CSF leak [1, 4, 5, 7, 8, 12]. In four of these patients, active interventions were performed, including lumbar subarachnoid drainage in three cases, transmastoid leak repair in one case, and transsphenoidal leak repair in one case which failed lumbar drainage. In in our case series, we note traumatic lumbar pneumorrhachis associated with basilar skull fractures, without evidence of CSF leak.

In the absence of an ongoing CSF leak, intradural air tends to resorb rapidly. No patient identified in our literature review required direct treatment for pneumorrhachis beyond those interventions aimed at traumatic brain injury and CSF leak. The majority of symptomatic pneumorrhachis reported in non-traumatic cases has been similarly managed conservatively. The application of high-flow oxygen, as is used in patients with pneumocephalus, is not specifically described in any case of pneumorrhachis. It was not used in either case presented here. Kennedy et al. described a patient with pressurized epidural air secondary to an analgesic catheter, which was decompressed by advancing a second epidural catheter into the entrapped locule of air, with immediate symptomatic improvement [18]. Uemura et al. described a case associated with a CSF fistula after intradural spinal tumor resection that resulted in symptomatic spinal cord compression, which ultimately required surgical evacuation and re-closure of the dura [20].

## Conclusions

Pneumorrhachis, or intraspinal air, is a rare finding after isolated head injury. In both cases in this report, as well as in the majority of prior reports, pneumorrhachis is associated with skull fractures allowing the entrainment of subarachnoid air. The intraspinal air *per se* is uncommonly symptomatic, but its presence should alert the clinician to the possibility of a CSF leak. All such patients should be carefully examined and observed for signs and symptoms of leak. Treatment is then directed at resolving the CSF fistula, as the air will absorb spontaneously.
